# Development and Application of a Semi quantitative Scoring Method for Ultrastructural Assessment of Acute Stress in Pancreatic Islets

**DOI:** 10.1097/TXD.0000000000001271

**Published:** 2021-12-16

**Authors:** Nicola J. Dyson, Nicole Kattner, Minna Honkanen-Scott, Bethany Hunter, Jennifer A. Doyle, Kathryn White, Tracey S. Davey, Rutger J. Ploeg, Yvonne A. Bury, Dina G. Tiniakos, James A. M. Shaw, William E. Scott

**Affiliations:** 1 Translational and Clinical Research Institute, Newcastle University, Newcastle upon Tyne, United Kingdom.; 2 Electron Microscopy Research Services, Newcastle University, Newcastle upon Tyne, United Kingdom.; 3 Nuffield Department of Surgical Science, John Radcliffe Hospital, Oxford, United Kingdom.; 4 Department of Cellular Pathology, Royal Victoria Infirmary, Newcastle upon Tyne Hospitals NHS Foundation Trust, Newcastle upon Tyne, United Kingdom.; 5 Department of Pathology, Aretaieion Hospital, Medical School, National and Kapodistrian University of Athens, Athens, Greece.; 6 Institute of Transplantation, Freeman Hospital, Newcastle upon Tyne Hospitals NHS Foundation Trust, Newcastle upon Tyne, United Kingdom.

## Abstract

**Background.:**

Pancreas and islet transplantation outcomes are negatively impacted by injury to the endocrine cells from acute stress during donor death, organ procurement, processing, and transplant procedures. Here, we report a novel electron microscopy scoring system, the Newcastle Pancreas Endocrine Stress Score (NPESS).

**Methods.:**

NPESS was adapted and expanded from our previously validated method for scoring pancreatic exocrine acinar cells, yielding a 4-point scale (0–3) classifying ultrastructural pathology in endocrine cell nuclei, mitochondria, endoplasmic reticulum, cytoplasmic vacuolization, and secretory granule depletion, with a maximum additive score of 15. We applied NPESS in a cohort of deceased organ donors after brainstem (DBD) and circulatory (DCD) death with a wide range of cold ischemic times (3.6–35.9 h) including 3 donors with type 1 and 3 with type 2 diabetes to assess islets in situ (n = 30) in addition to pancreata (n = 3) pre- and postislet isolation.

**Results.:**

In DBD pancreata, NPESS correlated with cold ischemic time (head: r = 0.55; *P* = 0.02) and mirrored exocrine score (r = 0.48; *P* = 0.01). When stratified by endocrine phenotype, cells with granules of heterogeneous morphology had higher scores than α, β, and δ cells (*P* < 0.0001). Cells of mixed endocrine-exocrine morphology were observed in association with increased NPESS (*P* = 0.02). Islet isolation was associated with improved NPESS (in situ: 8.39 ± 0.77 [Mean ± SD]; postisolation: 5.44 ± 0.31; *P* = 0.04).

**Conclusions.:**

NPESS provides a robust method for semiquantitative scoring of subcellular ultrastructural changes in human pancreatic endocrine cells in situ and following islet isolation with utility for unbiased evaluation of acute stress in organ transplantation research.

## INTRODUCTION

Insulin-dependent type 1 diabetes (T1D) is characterized by loss of endocrine β cells within the islets of Langerhans in the pancreas associated with autoimmunity.^1^ Transplantation of the vascularized whole pancreas or isolated islets can restore glycemic control in suitable recipients,^2-4^ although β-cell loss and failure to attain or maintain insulin independence are frequent occurrences following islet transplantation.^5^

Donor organs are subjected to multiple stresses during the peritransplant process, including stress associated with the following: death, trauma, intensive care management, organ procurement, and processing, which can adversely impact upon recipient outcomes.^6-10^ In particular, increased cold ischemia time (CIT) is associated with poorer isolated-islet yield and reduced graft survival.^11,12^

Various interventions and technologies have been developed to reduce or reverse the injuries to donor organs in the transplantation process, such as preservation solution additives, normothermic and hypothermic machine perfusion, and persufflation.^13-15^

Donor risk factors impacting graft success have been integrated into predictive scores for organ allocation^16^; however, we have previously shown that light microscopy analyses alone are insufficient to resolve the full extent of subcellular acute stress in the pancreas.^17^ A method for the quantitative electron microscopy (EM) examination of pancreatic endocrine cell stress would provide an invaluable addition to the current available techniques and aid in evaluating innovations to improve transplantation outcomes.

We have recently developed and validated the Newcastle Pancreatic Acinar Stress Score (NPASS), a novel scoring method for ultrastructural assessment of the acinar compartment of the pancreas.^17^ Here, we sought to extend this method to the endocrine pancreas and‚ in parallel‚ perform detailed subcellular characterization of islet cells across a range of donor organs and in isolated islets.

## MATERIALS AND METHODS

### Donor Organ Procurement and Biopsy Collection

Research was performed with written donor-relative consent in compliance with the UK Human Tissue Act of 2004 under specific ethical approvals by the UK Human Research Authority (05/MRE09/48 and 16NE0230).

A primary cohort of 30 deceased organ donors was selected to cover a broad spectrum of donor demographics and included 3 donors with T1D and 3 with type 2 diabetes (T2D) (Table 1). The cohort comprised organs with an intentionally wide range of CIT from 3.6 to 35.9 h and in donation after circulatory death (DCD) donors a range of warm ischemia time (WIT) from 9 to 103 min (Table 1) to facilitate the observation of the full spectrum of tissue changes. Tissue sampling was undertaken within the Quality in Organ Donation (QUOD) MRC-Expand program using established protocols.^17^ Analyses of isolated islets in comparison to the preisolation biopsy from the head of the pancreas were performed in an additional cohort of 3 donors with no history of diabetes (Table 2).

**Table 1.** Summary of donor characteristics

	Donor number	Donor type	History of diabetes	Age (y)	Gender	BMI (kg/m^2^)	CIT (h)	WIT (min)	Cause of death	Out of hospital cardiac arrest	ITU stay (d)	Number of cells scored	Regions scored
	**DBD1**	DBD	No	39	F	24.3	17.67		ICH	Yes	4	11	Tail
	**DBD2**	DBD	No	57	F	23.4	35.85		ICH	No	3	45	Head, tail
	**DBD3**	DBD	No	56	F	22.0	13.08		ICH	No	2	25	Tail
	**DBD4**	DBD	No	65	F	23.9	4.53		ICH	No	3	24	Tail
	**DBD5**	DBD	No	18	F	21.7	21.68		Hypoxic brain damage	Yes	6	25	Head
	**DBD6**	DBD	No	63	F	31.6	8.07		ICH	No	3	41	Head, tail
	**DBD7**	DBD	No	63	M	26.9	21.97		ICH	No	2	40	Head, tail
	**DBD8**	DBD	No	25	M	25.0	19.60		Hypoxic brain damage	Unknown	3	17	Tail
	**DBD9**	DBD	No	71	F	24.8	25.60		ICH	No	2	18	Tail
	**DBD10**	DBD	No	71	F	26.3	3.55		Hypoxic brain damage	No	1	49	Head, tail
	**DBD11**	DBD	No	63	F	30.8	7.32		ICH	Yes	2	25	Tail
	**DBD12**	DBD	No	29	F	25.4	5.48		Hypoxic brain damage	Yes	4	25	Head
	**DBD13**	DBD	No	73	F	33.8	17.20		ICH	Yes	1	25	Tail
	**DBD14**	DBD	No	56	F	23.4	8.68		ICH	No	2	25	Head
	**DBD15**	DBD	No	18	F	41.5	19.10		Hypoxic brain damage	Yes	4	13	Tail
	**DBD16**	DBD	T1D	38	M	23.2	4.65		Trauma	No	2	50	Head, tail
	**DBD17**	DBD	T1D	33	F	21.6	6.53		ICH	No	3	24	Tail
	**DBD18**	DBD	T1D	60	F	26.7	7.68		ICH	No	2	25	Tail
	**DBD19**	DBD	T2D	70	F	34.9	6.98		ICH	No	8	13	Head
	**DBD20**	DBD	T2D	63	F	32.8	5.03		ICH	No	3	25	Tail
	**DBD21**	DBD	T2D	71	M	28.6	6.28		ICH	No	2	37	Head, tail
**DBD no diabetes**	Mean			51		27.0	15.29				2.8		
	Range			18–73		21.7–41.5	3.55–35.85				1–6		
	Summary				13 (87%) female				10 (67%) ICH	6 (40%) OHCA			
**DBD T1D**	Mean			44		23.8	6.29				2.3		
	Range			33–60		21.6–26.7	4.65–7.68				2–3		
	Summary				2 (67%) female				2 (67%) ICH	0 (0%) OHCA			
**DBD T2D**	Mean			68		32.1	6.10				4.3		
	Range			63–71		28.6–34.9	5.03–6.98				2–8		
	Summary				2 (67%) female				3 (100%) ICH	0 (0%) OHCA			
**DBD all**	Mean			52		27.3	15.29				3.0		
	Range			18–73		21.6–41.5	3.55–35.85				1–8		
	Summary		15 (71%) non diabetic		17 (81%) female				15 (71%) ICH	6 (29%) OHCA			
	**DCD1**	DCD	No	55	M	26.6	26.48	9	Hypoxic brain damage	Yes	5	16	Tail
	**DCD2**	DCD	No	27	M	37.2	22.80	20	Trauma	No	4	25	Head
	**DCD3**	DCD	No	62	M	26.9	8.35	25	ICH	No	2	50	Head, tail
	**DCD4**	DCD	No	36	M	27.8	12.90	103	Hypoxic brain damage	Yes	16	24	Head
	**DCD5**	DCD	No	54	F	39.0	3.97	n/a	ICH	No	31	44	Head, tail
	**DCD6**	DCD	No	56	F	27.5	18.60	32	Respiratory failure	No	10	31	Head, tail
	**DCD7**	DCD	No	59	M	18.8	4.48	63	Hypoxic brain damage	Yes	1	39	Head, tail
	**DCD8**	DCD	No	32	M	22.2	4.12	75	ICH	No	9	25	Head
	**DCD9**	DCD	No	42	M	33.0	7.58	89	ICH	No	2	50	Head, tail
**DCD all**	Mean			47		28.8	12.14	52			8.9		
	Range			27–62		18.8–39	3.97–26.48	9–103			1–31		
	Summary		9 (100%) non diabetic		2 (22%) female				4 (44%) ICH	3 (33%) OHCA			

BMI, body mass index; CIT, cold ischemia time; DBD, donation after brainstem death; DCD, donation after circulatory death; ICH, intracranial hemorrhage; ITU, intensive therapy unit; WIT, warm ischemia time.

**Table 2.** Summary of donor characteristics for isolated islets

	Donor type	History of diabetes	Age (y)	Gender	BMI (kg/m^2^)	CIT (h)	WIT (min)	Cause of death	Out of hospital cardiac arrest	ITU stay (d)	Number of cells scored (tissue)	Number of cells scored (D0)	Number of cells scored (D1)	Islet purity (%)	Islet viability (%)	Islet yield (IEQ)	IEQ/tissue gram	IEQ/gram of digested tissue
**I-1**	DCD	No	49	F	26.6	13.10	12	Hypoxic brain damage	Yes	6	18	25	25	60	78	152,489	1897	3546
**I-2**	DCD	No	65	F	28.2	6.05	16	ICH, COPD	No	18	14	25	25	80	69	202,316	2136	2738
**I-3**	DBD	No	37	M	20.5	5.57		Hypoxic brain damage	No	2	16	25	25	35	82	132,561	2052	4983

D0 islets were sampled 1 to 2 h after being placed in culture; D1 islets were sampled 12 to 18 h after being placed in culture. Islet viability was assessed by propidium iodide staining on D1.^21^

BMI, body mass index; CIT, cold ischemia time; COPD, chronic obstructive pulmonary disease; DBD, donation after brainstem death; DCD, donation after circulatory death; ICH, intracranial hemorrhage; IEQ, islet equivalent number; ITU, intensive therapy unit; WIT, warm ischemia time.

Donor pancreata were procured by the UK National Organ Retrieval Service utilizing standardized procedures. Pancreas dissection was performed in a standardized manner in a 4°C cold room, as previously described,^17^ and tissue was sampled from each of 8 anatomic regions of the pancreas (P1–P8), with P1 corresponding to the head/uncinate process and subsequent blocks moving incrementally toward the tail region (P8). Samples were stained with dithizone (Merck; Gillingham, United Kingdom) to distinguish islets within tissue, microdissected into 1 to 2 mm^3^ islet-rich biopsies, and fixed in 2% glutaraldehyde in 0.1 M sodium cacodylate buffer (Agar Scientific; London, United Kingdom). Optimizations confirmed that dithizone staining had no impact on downstream islet imaging.

### Islet Isolation

Islet isolations were performed using a modified Ricordi method.^18-20^ Briefly, the pancreas was perfused with collagenase and neutral protease (PELOBiotech; Munich, Germany), with islet dissociation carried out in a Ricordi chamber (Biorep; Miami, FL), followed by density gradient centrifugation in a COBE 2991 processor (Terumo; Shibuya, Japan). A single tissue biopsy was taken from the head of the pancreas upon cannulation of the main pancreatic duct before commencing enzyme perfusion. Following isolation, islets were cultured in CMRL (Corning Life Sciences; Tewksbury, MA) supplemented with 0.5% human serum albumin (BioIVT; London, United Kingdom), Hepes (Merck; Gillingham, United Kingdom), L-glutamine‚ and penicillin/streptomycin (Fisher Scientific; Loughborough, United Kingdom). Islets were sampled 1 to 2 h after being placed in culture (D0) and 12 to 18 h postisolation (D1).

### Transmission EM

Glutaraldehyde-fixed tissue specimens were postfixed with osmium tetroxide, dehydrated in acetone, and embedded in epoxy resin (TAAB; Aldermaston, United Kingdom). Seventy nanometer ultrathin sections were obtained using the Ultracut E (Reichert; Vienna, Austria), mounted on pioloform-coated Cu-grids, and poststained with uranyl acetate and lead citrate. Images were acquired on the Hitachi HT7800 120 kV transmission electron microscope (Hitachi High-Technologies; Abingdon, United Kingdom).

One section from the head (P1) and 1 from the tail (P8) of the pancreas were imaged for each donor. The whole section was scanned for presence of endocrine‚ tissue and, when present, endocrine cells were assessed at random up to a maximum of 25 cells. If fewer than 25 endocrine cells were present, a minimum of 10 cells was set as the cutoff for inclusion in the cohort. If more than 1 islet was present, cells were selected from all islets. A single image of each cell was captured at 4000 to 6000× magnification depending on cell size. This method was also applied to isolated-islet imaging. A single operator experienced in the development and application of NPASS^17^ scored all samples.

The NPASS criteria use a scale from 0 to 3 to assess acute stress in acinar cells, with 0 representing normal appearance and 3 representing the most severe damage. Four subcellular compartments were assessed: nuclear chromatin condensation, mitochondrial swelling, endoplasmic reticulum (ER) dilation, and vacuolization.^17^ Initial evaluation of the severity of the ultrastructural changes in the endocrine cells was performed in alignment with these criteria. This revealed that a similar breadth of ultrastructural pathological changes was present in the endocrine cell organelles, and‚ therefore‚ these 4 categories were retained for the semiquantitative assessment of pancreatic islet cells (Table 3).

**Table 3.** NPESS criteria for scoring of pancreatic endocrine cells

Degradation score	Nucleus	Mitochondria	ER dilation	Vacuolization	Cytoplasmic vesicle depletion
0	Normal appearance	Normal appearance	Absent	Absent	Absent
1	Chromatin condensation or loss (mild to moderate)	Open appearance	Mild dilation	Mild vacuolization(0%–25% of cell)	Mild vesicle loss (<25% of cell membrane)
2	Chromatin condensation or loss (moderate to severe)	Swollen appearance	Moderate dilation	Moderate vacuolization (25%–50% of cell)	Moderate vesicle loss (25%–50% of cell membrane)
3	Chromatin condensation and detachment from nuclear membrane	“Blown” mitochondria—disrupted cristae, loss of highly recognizable components	Severe dilation	Severe vacuolization (>50% of cell)	Severe vesicle loss (>50% of cell membrane)

ER, endoplasmic reticulum; NPESS, Newcastle Pancreas Endocrine Stress Score.

### Statistics

Statistical analyses were carried out using Prism version 8 (GraphPad Inc; San Diego, CA). Two-tailed Spearman’s r was used to test correlations. Differences between means in the Quality in Organ Donation cohort were calculated with 2-tailed paired/unpaired Student’s *t* tests or 1-way ANOVA with Tukey’s multiple comparisons test. Repeated measures 1-way ANOVA with Tukey’s multiple comparisons test was used in the isolation cohort. Fisher’s exact test was performed to assess cell-type proportions in different donor groups. Data are reported as mean (± SD). Statistical significance was defined as *P *<* *0.05.

## RESULTS

### Evaluation of Endocrine Ultrastructure

**FIGURE 1.**
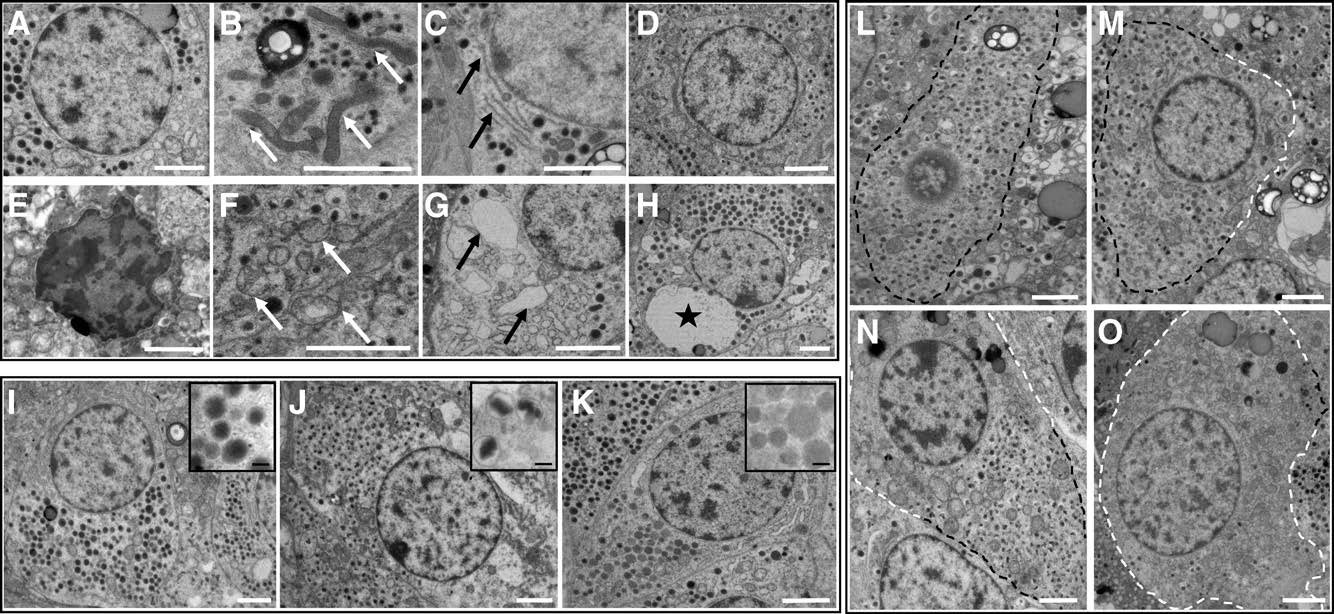
Representative TEM images of endocrine cells. A–D, These images show normal morphology of cells and organelles. E–H, These show example images of acute stress. Nucleus (A, E), mitochondria (B, F, white arrows), endoplasmic reticulum (C, G, black arrows), vacuolization (D, cell with no vacuolization present) (H, black star indicates large vacuole). Cells in A, C, G, and H are α cells; cells in B, D, E, and F are β cells. I–K, Representative images of endocrine cell types: I, α cell; J, β cell; K, δ cell. Inset boxes show the morphology of the respective endocrine granules. Vesicle depletion is scored according to the loss of endocrine granules at cell membranes: L, score 0; M, score 1; N, score 2; O, score 3. Black dashed line indicates area with vesicles; white dashed line indicates depleted area. All cells in L through O are β cells. White scale bars: 2 µm. Black scale bars: 200 nm. TEM, transmission electron microscopy.

Nuclei were similar in appearance to those in exocrine cells, with chromatin evenly distributed in healthy cells (Figure 1A) and chromatin clumping apparent following acute stress (Figure 1E).

Mitochondria were abundant and small in size (0.075– 0.6 µm^2^) relative to those in acinar cells (0.2–0.9 µm^2^). Healthy mitochondria had an elongated ovoid shape (Figure 1B); under acute stress‚ this altered to a rounded, more electron-lucent appearance, with swelling and destruction of mitochondrial cristae (Figure 1F).

The ER was recognized by parallel electron-dense lines in close proximity, generally surrounding a more electron-lucent interior and joined at the ends to form cisternae. The ER was often sparsely visible in endocrine cells‚ but in the majority of cases‚ ER cisternae could still be evaluated (Figure 1C). Ribosomes were occasionally visible at a higher magnification. When heavily dilated, the ER appeared as irregularly shaped areas of low electron density (Figure 1G).

Vacuoles appeared in cells as circular areas of low electron density and could be distinguished from ER dilation by their rounded shape and lack of ribosomes (Figure 1H).

Endocrine granules were present throughout the interior of the cell and at the cell membranes (Figure 1). Endocrine cell type was identified by granule morphology: α cells had uniformly circular, electron-dense granules (Figure 1I); β cells could be identified by the characteristic halo surrounding smaller electron-dense granules‚ which were heterogeneous in shape (Figure 1J); δ-cell granules were similar in size to glucagon granules but with a more variable electron-lucent density (Figure 1K). Only a single morphological PP cell was observed in the whole cohort: the PP granules were rounded and electron-dense but approximately half the size (100–200 nm) of α-cell glucagon granules.

Loss of endocrine granules, particularly at the cell membrane, was frequently observed (Figure 1L–O). The extent of loss varied from mild, with a minority of the cell area affected, to severe, in which only a small number of scattered vesicles remained. This observation led to the addition of a further scoring category of endocrine vesicle depletion, defined according to the percentage of vesicle loss from the cell membrane (Table 3). This modified scoring system for endocrine cells of the pancreas will subsequently be referred to as the Newcastle Pancreas Endocrine Stress Score (NPESS).

### NPESS Scores in a Cohort of Deceased Organ Donors

Analysis of pancreatic head and tail regions in a cohort of 30 deceased donors (Table 1) showed mitochondrial swelling in all donors, with ubiquitously high scores (mean‚ 2.73; range‚ 2.1–3.0)‚ even with a short CIT of 4 h. In contrast, nuclear scores to be low (mean‚ 0.79; range‚ 0.16–2.25). The widest range of scores (0.3–2.75) was seen in the ER. Evidence of at least mild endocrine vesicle depletion was ubiquitous, with a mean score of 2.17 (range‚ 1.32–2.83).

**FIGURE 2.**
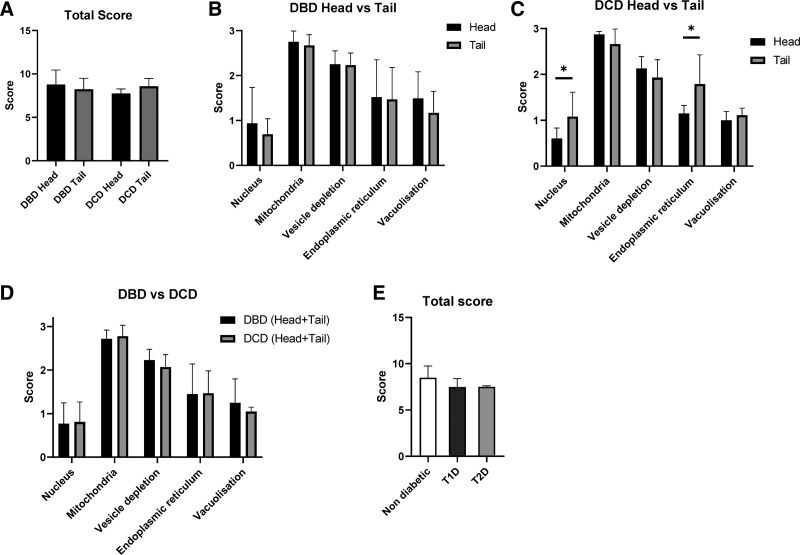
Comparison of NPESS between head and tail of pancreas in DBD and DCD donors and by diabetes status. A, Total NPESS in DBD head/tail and DCD head/tail. B, NPESS subscores in head (n = 10) and tail (n = 17) of pancreas in DBD donors. C, NPESS subscores in head (n = 8) and tail (n = 6) of pancreas in DCD donors. D, Total (head + tail) NPESS in DBD (n = 21) versus DCD (n = 9) donors. E, Total score in donors without established diabetes (n = 21), T1D (n = 3), and T2D (n = 3). Bars represent mean ± SD (*P < 0.05). DBD, donors after brainstem death; DCD, donors after circulatory death; NPESS, Newcastle Pancreas Endocrine Stress Score.

Total NPESS scores were comparable between the head and tail of pancreata in both donation after brainstem death (DBD) and DCD donors (Figure 2). Head versus tail subscales were also comparable despite statistically significant differences in the nucleus and ER scores in DCDs, which was likely due to a type I error in view of the smaller sample size compared with DBDs.

In the wide range of donors within the cohort, no differences in mean scores were seen between DBD and DCD (Figure 2). The cohort included 3 DBD donors with T1D and 3 with T2D. Total NPESS scores were comparable with those without diabetes (Figure 2).

**FIGURE 3.**
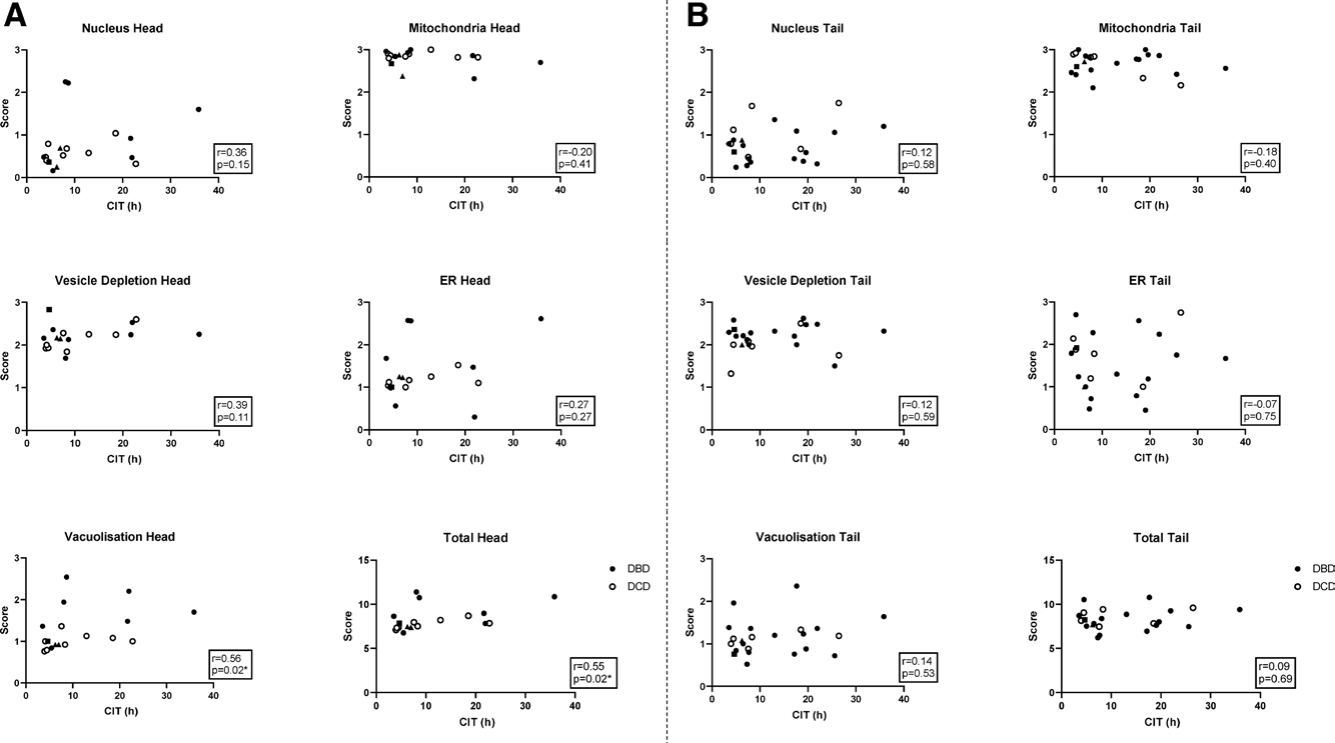
Comparison between NPESS and cold ischemia time. Scores in DBD donors (black circles) and DCD donors (white circles) are plotted against CIT in pancreas head (A) and pancreas tail (B). Donors with T1D and T2D are represented by squares and triangles, respectively. CIT, cold ischemia time; DBD, donors after brainstem death; DCD, donors after circulatory death; NPESS, Newcastle Pancreas Endocrine Stress Score.

There was a significant correlation between CIT and overall NPESS in the head of the pancreas but not in the pancreatic tail (Figure 3). Correlation between CIT and individual NPESS subscale parameters in the head of the pancreas only reached statistical significance for the vacuolization score (Figure 3A). Subscale NPESS parameters within the tail of the pancreas did not correlate with CIT (Figure 3B). Although numbers of donors with T1D and T2D were too small for statistical comparison testing, all were DBD donors with a relatively short CIT and did not appear to be outliers from the overall cohort for any of the NPESS parameter scores (Figure 3). There was no association of any score with WIT in DCD donors (data not shown).

### Comparison of NPESS With NPASS in a Cohort of Deceased Organ Donors

**FIGURE 4.**
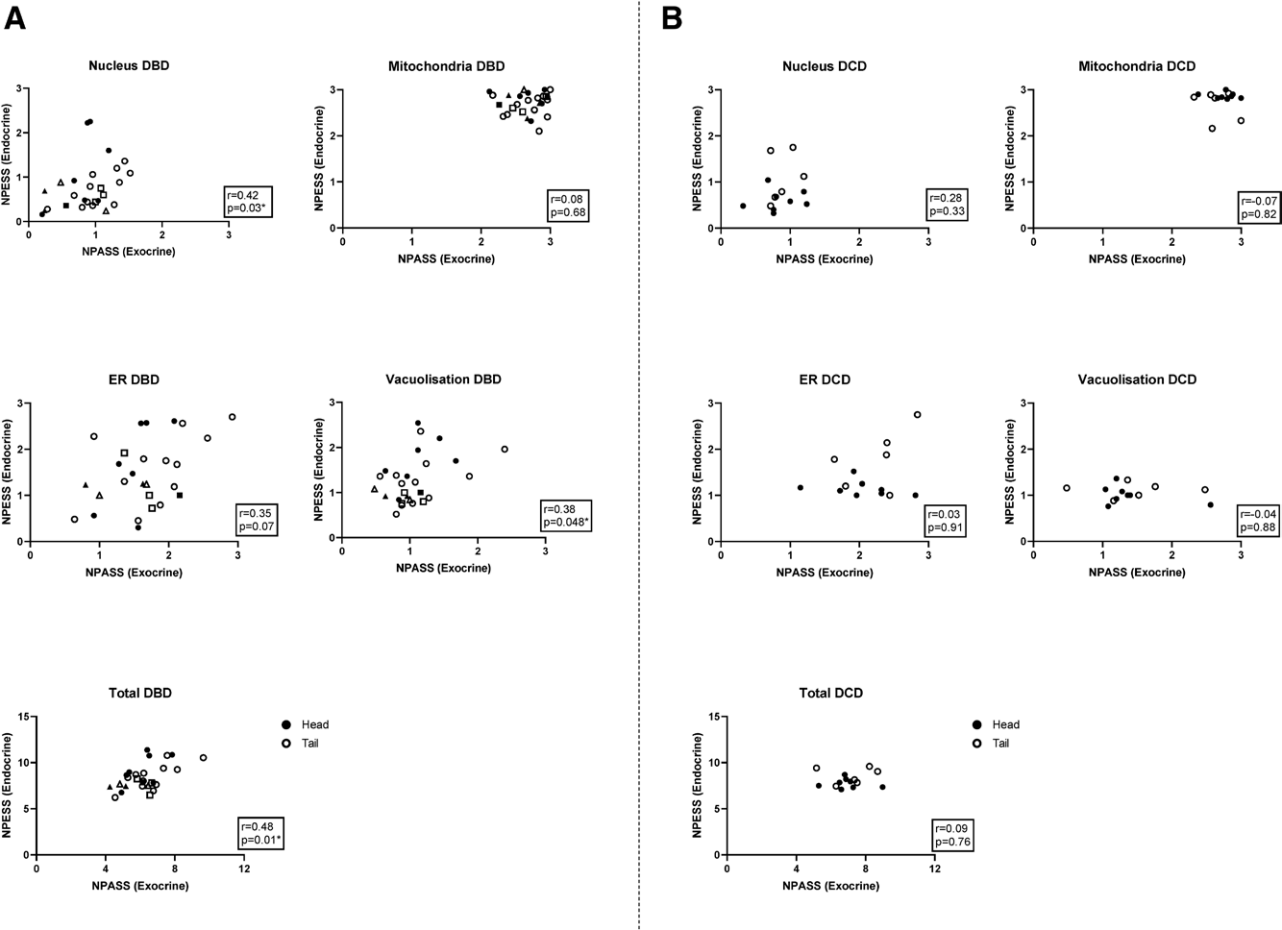
Correlations of NPESS (endocrine) scores with NPASS (exocrine) scores. NPASS is plotted against NPESS for each specimen analyzed, with pancreas head and pancreas tail denoted by black and white symbols, respectively. Correlation plots are shown for individual organelle and total scores in DBD (A) and DCD donors (B). Donors with T1D and T2D are represented by squares and triangles, respectively. DBD, donors after brainstem death; DCD, donors after circulatory death; NPASS, Newcastle Pancreatic Acinar Stress Score.; NPESS, Newcastle Pancreas Endocrine Stress Score.

In parallel with NPESS quantification, NPASS scoring (according to published methods) was performed on exocrine tissue within each section. Mirroring NPESS scores, and as previously published,^17^ NPASS mitochondrial scores were ubiquitously high‚ with nuclear scores tending to be low and ER scores having the widest variation between donors. Plots of NPASS versus NPESS demonstrated comparable organelle stress in acinar and endocrine cells within biopsies obtained from the head and tail of pancreata in DBD donors (Figure 4A). Again, deceased donors with known diabetes were not outliers, indicative of comparable subcellular organelle ultrastructural morphology in both endocrine and acinar cells. There were no significant correlations between endocrine and acinar stress scores in the smaller number of DCD donors evaluated (Figure 4B).

### EM Identification and Analysis of Individual Endocrine Phenotypes

**FIGURE 5.**
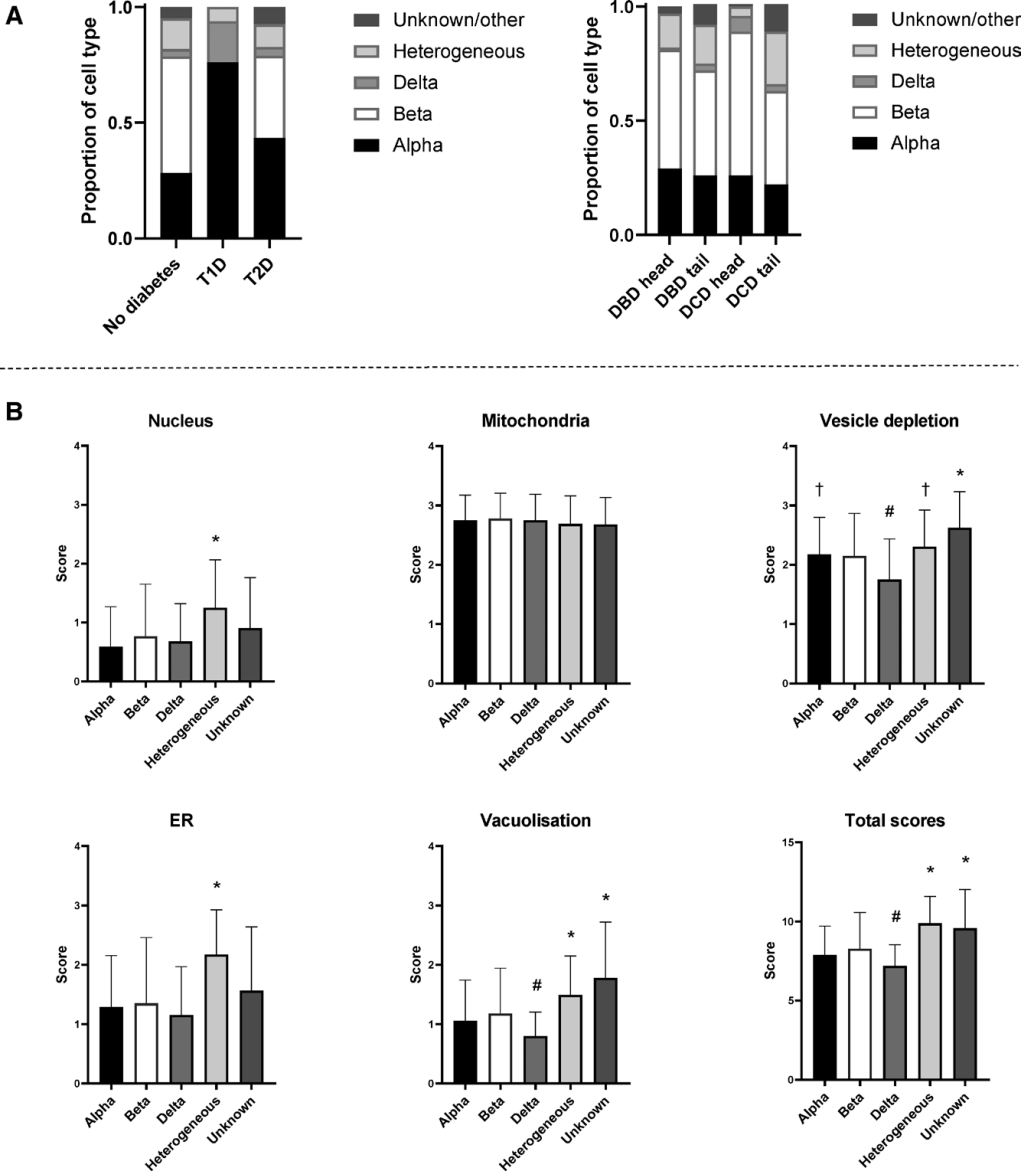
Analysis of individual endocrine phenotypes. A, Overall proportions of individual cell types in the cohort, stratified by diabetes status, donor type, and pancreas region. B, NPESS scores for individual cells stratified by cell type. Bars represent mean ± SD. *P < 0.05 versus α-, β-, and δ cells. #*P* < 0.05 versus β cells. †*P* < 0.05 versus δ cells. n = 316 (α), n = 369 (β), n = 44 (δ), n = 124 (heterogeneous), and n = 32 (other/unknown). ER, endoplasmic reticulum; NPESS, Newcastle Pancreas Endocrine Stress Score.

In the donors without diabetes, proportions of α, and δ cells (identified by the morphological appearance of granules) were broadly in line with previously reported frequencies (28% α, 50% β, 3% δ) (Figure 5A, left panel).^22^ No β cells were observed in any of the T1D donors, and α cells predominated over δ cells (78% versus 18%). Proportions of α cells and β cells were comparable in T2D donors (43% α, 36% β, 4% δ) and β cell to α cell ratio was significantly lower in T2D compared with donors without diabetes (*P* = 0.005). Occasionally, cells containing granules that resembled those of more than 1 cell type, typically α and β, were observed (16% of cells). There was no significant difference in the prevalence of these heterogeneous cells in donors with/without diabetes. In severe cases of vesicle depletion, the lack of endocrine vesicles prevented identification of the cell type (4%, classified as unknown/other). No significant differences in cell-type proportions were detected when the head versus tail of pancreata or DBD versus DCD were compared across the whole cohort (Figure 5A, right panel).

When NPESS for individual cells across the whole cohort, including donors with and without diabetes, were stratified by endocrine cell type, cells defined as heterogeneous with more than 1 type of endocrine granule had significantly higher subscale and total stress scores, with the exception of the mitochondrial score‚ which was consistently high in all cell phenotypes (Figure 5B). This persisted when donors with diabetes were omitted (Figure S1, SDC, http://links.lww.com/TXD/A397). Significantly lower vesicle depletion and vacuolization leading to lower overall NPESS were seen in δ cells relative to β cells. When donors with diabetes were omitted, only the difference in vesicle depletion scores remained significant. NPESS scores in α and β cells were comparable, including and excluding donors with diabetes.

### Intermediate Endocrine-Exocrine Cells

A small number of cells exhibited features of both endocrine and acinar morphology (Figure S2, SDC, http://links.lww.com/TXD/A397). These were identified by the presence of zymogen granules in addition to endocrine granules. Zymogen granules are similar in electron density to glucagon granules but are larger in size, with a diameter of 300 to 900 nm versus 200 to 400 nm. These mixed-phenotype “intermediate cells” were identified in 9 of 30 donors (30%) but were infrequent‚ with only 1 to 3 cells counted in each donor. Intermediate cells were distributed equally across both the head and tail of the pancreas, generally located toward the islet periphery or as isolated endocrine cells adjacent to acinar cells, and included α cells, β cells, and cells of ambiguous phenotype. One intermediate cell was present in D1 free islets from donor I-2. Demographic analysis of the donors with intermediate cells in the islets revealed no significant association with diabetes status, and although trends toward a greater age and lower body mass index were observed, these were not statistically significant. No further demographic associations were apparent. Donors with intermediate cells had significantly increased NPESS scores (*P* = 0.02) (Figure S2, SDC, http://links.lww.com/TXD/A397). No association was observed with NPASS score (data not shown).

### Impact of Islet Isolation on NPESS

**FIGURE 6.**
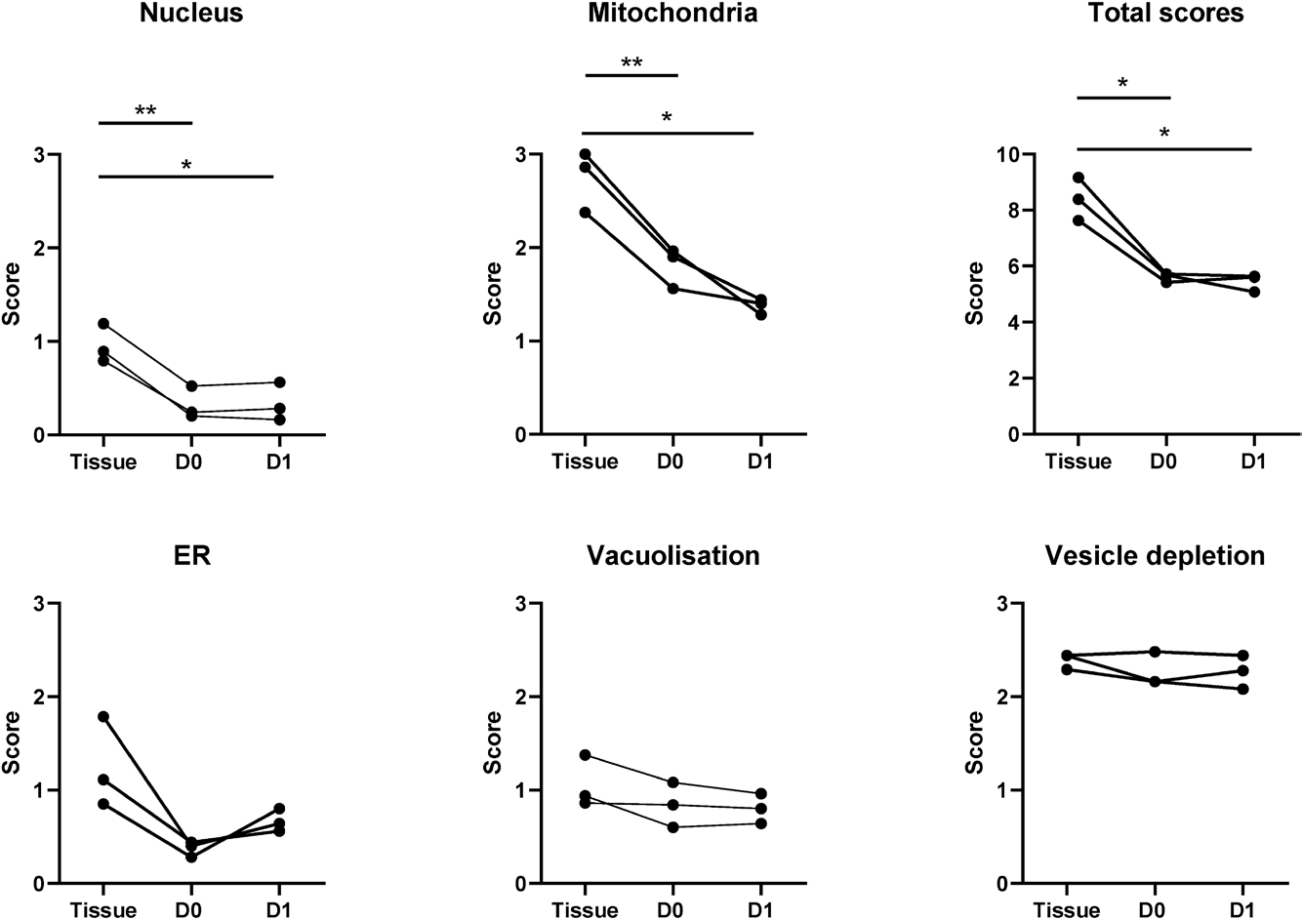
Isolated islets have a reduced stress phenotype when compared with matched preisolation islets in situ. Scores for individual organelles and total scores of preisolation tissue and islets at day 0 and day 1 in culture from 3 donor organs are shown. D0 islets were sampled 1 to 2 h after being placed in culture; D1 islets were sampled 12 to 18 h after being placed in culture. *P < 0.05, **P < 0.01. ER, endoplasmic reticulum.

Islets from 3 additional donor pancreata (2 DCD; 1 DBD) were fixed and analyzed both 1 to 2 h postisolation and following 12 to 18 h in culture in comparison with tissue biopsies collected before enzyme perfusion. Total NPESS scores were significantly lower in isolated islets than intact pancreata, with significant improvements in appearances of all organelles but unresolved endocrine vesicle depletion (Figure 6).

## DISCUSSION

Building on a recently validated method for standardized EM evaluation of human pancreatic acinar cells, we have developed a robust semiquantitative scoring system for systematic evaluation of subcellular organelles in human pancreatic endocrine cells within the intact organ and isolated islets. Application within a cohort of deceased donor whole pancreata showed comparable scores between DBD and DCD donors and similar endocrine and acinar stress scores in pancreatic head and tail biopsies. P1 endocrine stress scores in donors with or without diabetes correlated with CIT and were comparable to NPASS. NPESS has additional potential for subcellular morphological characterization of isolated islets.

Ultrastructural alterations including nuclear chromatin condensation, mitochondrial swelling, ER dilation and vacuolization have previously been reported in islet cells undergoing ischemic stress.^23-26^ Loss of endocrine vesicles has been demonstrated by EM in human islets following exposure to acinar cell proteases^27^ and in rat pancreas in a study of cyclosporine A-induced injury.^28^ More recently, quantitative scoring of EM changes has been used to assess cellular injury in a rodent model of streptozotocin-induced diabetes.^29^ Unbiased assessment of pancreatic endocrine cell ultrastructural appearance in deceased donor organs within the current study has demonstrated the range of morphological appearances in the nucleus, mitochondria, ER, cytoplasmic vacuolization, and secretory granules enabling development of a novel semiquantitative scoring system comprising subscales for each of these parameters and a total score (NPESS).

The utility of NPESS to quantify ultrastructural changes across a broad spectrum of organ donors including a wide CIT and WIT range has been confirmed showing comparable scores in DBD and DCD pancreata. Inclusion of a small number of donors with T1D and T2D showed that these were not outliers, suggesting that NPESS is more an indicator of acute subcellular stress than chronic cellular dysfunction.

Mitochondrial scores were high in all donors‚ including both DBD and DCD donors (all of which were optimally retrieved following clinical standard operating procedures).^30^ In contrast, nuclear scores were low and ER scores were most variable between organs. We have previously reported this organelle-specific pattern in pancreatic acinar cells.^17^ In addition, endocrine secretory vesicle depletion was evident in all donors. NPESS and NPASS scores‚ including subscales‚ were closely matched in the current study within individual organs and head/tail tissue blocks. These findings support common pathways through which ischemia affects acinar and endocrine cells and comparable susceptibility of these different pancreatic cell phenotypes.

CIT is a negative predictor of islet isolation outcomes and 90-d vascularized pancreas graft survival.^12,31^ Pancreatic head but not tail NPESS correlated with CIT in this analysis. Differences in correlations between the 2 pancreatic regions analyzed may reflect the anatomical differences in the pancreas, with the head deriving from the ventral bud during pancreas development, whereas the body and tail derive from the dorsal bud^32^; however, this study was designed for methodological development and validation and not powered to confirm associations; therefore‚ correlations were not absolute. Given the complexity of factors impacting upon donor organs, extended studies on larger cohorts will be necessary for sufficient power to clarify the effect of single variables and validate these hypotheses.

Identification of individual endocrine phenotypes was undertaken by morphological analysis. Although absence of confirmation of cell type by immunogold-hormone labeling was a potential weakness of the current study, an a priori decision was made to maximize ultrastructural preservation through osmium tetroxide fixation despite its negative impact on tissue protein antigenicity.^33,34^ Accuracy of identification was supported by absence of detected β cells in donors with T1D and increased α-cell to β-cell ratio in T2D.

Although reliance upon aerobic versus anaerobic glycolysis^35^ and vulnerability to oxidative stress with reduced antioxidant enzyme expression^36^ in β cells in comparison with α cells in vitro have been reported, in situ NPESS scores were comparable in α and β cells. Although preliminary, the current data suggest that δ cells may be more resistant to ultrastructural damage associated with organ donation. Whether this reflects their distinct polygonal morphology with neuron-like cytoplasmic projections^22,37^ requires further elucidation.

Bihormonal cells expressing both insulin and glucagon have been described particularly in association with T2D and T1D.^38-41^ Although there was no association with known diabetes in the current study, we observed a trend toward higher α-cell to β-cell ratios in donors with a high percentage of cells with heterogeneous appearance (r = 0.36; *P* = 0.067). Furthermore, these cells appeared sensitive to stress associated with organ donation‚ which had highest NPESS scores of all cell types. Without antibody-staining confirmation of bihormone expression, it cannot be ruled out that the heterogeneous appearance of the granules may be due to the impact of acute stress on normal granule morphology or the loss of defined cell membranes in severely degraded cells, impeding cell distinction.

Intermediate endocrine-exocrine cells have been previously reported in both the exocrine and endocrine pancreata.^42,43^ One study identified cells containing both zymogen-like and insulin-like granules in T2D noting close proximity to macrophages and mast cells.^44^ Another found a greater prevalence of intermediate cells in T1D and autoantibody-positive donors in addition to evidence of ER dilation and mitochondrial damage in these cells.^45^ Intermediate cells were identified in donors with and without diabetes in the current study, and no immune cell infiltration was observed in the vicinity of these cells. Presence of intermediate cells was associated with a higher NPESS score within the endocrine compartment‚ suggesting the possibility that this phenotype may be induced by the stress associated with organ donation or from transdifferentiation resulting from chronic stress, which may increase susceptibility to acute stress.

The process of islet isolation is associated with additional enzymatic, chemical, and mechanical stress leading to the loss of basement membrane, the induction of mitogen-activated protein kinase and nuclear factor κ-B stress signaling pathways, and the poly ADP-ribose polymerase activation of apoptotic and necrotic pathways.^46-48^ Pilot data demonstrating the utility of NPESS in assessing isolated islets, however, showed consistently reduced NPESS scores in comparison with islets within the donor pancreas before isolation. Despite the negative impact of increased CIT on islet isolation outcomes, viable islets suitable for transplantation can still be obtained from high CIT organs.^31,49^ All 3 isolations in this study had a similar pattern of “recovery” of the islets postisolation from donors with a CIT range of 5.6 to 13.1 h. The natural selection for healthier cells imposed by the isolation process may be an important factor accounting for this effect, although this may also demonstrate repair of ischemic damage following restoration of oxygenation. The difference in the mitochondria is particularly striking‚ with scores of 1.28 to 1.96 in the isolated islets substantially lower than those seen in all whole pancreata within this study. During the ischemic period of ischemia-reperfusion injury, hypoxia and reduced ATP results in electrolyte imbalance due to failure of ATPase pumps, causing cell and mitochondrial swelling. Following restoration of oxygen‚ generation of ROS in the mitochondria induces oxidative stress that can result in cell death.^50^ The reduced mitochondrial swelling observed in isolated islets with an absence of signs of injury in other organelles may indicate recovery in culture with the removal of damaged mitochondria via mitophagy.^51,52^ It is established that restoration of oxygen results in ischemia-reperfusion injury driven by rapid conversion of accumulated succinate to fumarate. This process may lead to a number of changes that could resolve over the 1-d postisolation recovery period‚ including apoptosis of critically damaged cells and autophagic removal of damaged organelles.^50-52^

It should be noted that these are pilot data from a small number of islet isolations, and additional studies on free islets are needed to strengthen these observations. In particular, functional studies including oxygen consumption rate, glucose-stimulated insulin secretion‚ and analyses of apoptotic and necrotic pathways may shed further light on the cellular mechanisms underlying the NPESS, and we plan to follow up with these analyses in further work. Additionally, retrospective NPESS scoring on pretransplant islet preparations would enable the study of correlations with clinical outcomes.

Impacts of islet culture on islet cells, both deleterious and beneficial, have been described with the upregulation of proinflammatory and stress-induced genes^53,54^ and islet attrition, yet improved morphology and viability.^55^ Determination of the optimal temperature and duration for islet culture before transplantation is an area of ongoing research,^56^ and the NPESS may provide an additional tool for researchers in evaluating and optimizing islet culture. NPESS could be implemented in the evaluation of agents that may impact islet stress and function, such as free fatty acids or nicotinamide.^57,58^ NPESS also has utility for in vivo transplantation studies.

The original NPASS system was developed as a tool to assess (sub) cellular stress impacting the acinar cells of the pancreas. Here, we demonstrate that an adapted version of this method can be applied to endocrine cells in conjunction with the exocrine score for comprehensive assessment of pancreata or as a stand-alone tool for specific investigation of islet stress both in situ and in isolated islets. The NPESS has utility for retrospective analysis of donor tissue/islets following clinical transplantation and for prospective preclinical studies. Application in experimental models will facilitate a deeper understanding of how islet cells are impacted by ischemia and by interventions to mitigate this, ultimately enhancing clinical outcomes.
